# *In Vivo* Characterization of Neutrophil Extracellular Traps in Various Organs of a Murine Sepsis Model

**DOI:** 10.1371/journal.pone.0111888

**Published:** 2014-11-05

**Authors:** Koji Tanaka, Yuhki Koike, Tadanobu Shimura, Masato Okigami, Shozo Ide, Yuji Toiyama, Yoshinaga Okugawa, Yasuhiro Inoue, Toshimitsu Araki, Keiichi Uchida, Yasuhiko Mohri, Akira Mizoguchi, Masato Kusunoki

**Affiliations:** 1 Departments of Gastrointestinal and Paediatric Surgery, Mie University Graduate School of Medicine, Tsu, Mie, Japan; 2 Departments of Neural Regeneration and Cell Communication, Mie University Graduate School of Medicine, Tsu, Mie, Japan; Maastricht University, Netherlands

## Abstract

Neutrophil extracellular traps (NETs) represent extracellular microbial trapping and killing. Recently, it has been implicated in thrombogenesis, autoimmune disease, and cancer progression. The aim of this study was to characterize NETs in various organs of a murine sepsis model *in vivo* and to investigate their associations with platelets, leukocytes, or vascular endothelium. NETs were classified as two distinct forms; cell-free NETs that were released away from neutrophils and anchored NETs that were anchored to neutrophils. Circulating cell-free NETs were characterized as fragmented or cotton-like structures, while anchored NETs were characterized as linear, reticular, membranous, or spot-like structures. In septic mice, both anchored and cell-free NETs were significantly increased in postcapillary venules of the cecum and hepatic sinusoids with increased leukocyte-endothelial interactions. NETs were also observed in both alveolar space and pulmonary capillaries of the lung. The interactions of NETs with platelet aggregates, leukocyte-platelet aggregates or vascular endothelium of arterioles and venules were observed in the microcirculation of septic mice. Microvessel occlusions which may be caused by platelet aggregates or leukocyte-platelet aggregates and heterogeneously decreased blood flow were also observed in septic mice. NETs appeared to be associated with the formation of platelet aggregates or leukocyte-platelet aggregates. These observational findings may suggest the adverse effect of intravascular NETs on the host during a sepsis.

## Introduction

Neutrophil extracellular traps (NETs) are known to be part of an antimicrobial defense system. They are released from neutrophils activated by phorbol myristate acetate, interleukin-8, lipopolysaccharide (LPS), and various pathogens [1]. They exhibit fibrous mesh-like, web-like, or string-like structures and are composed of DNA, histones, and granule proteins such as neutrophil elastase or myeloperoxidase [2]. At present, NETs research focused on not only exploring its physiological role [3], [4], but also its pathophysiological relevance in various diseases including thrombogenesis [5], [6], atherosclerosis [7], [8], autoimmune disease [9], [10], and cancer metastasis [11], [12]. In addition to the function of extracellular bacterial trapping and killing, the adverse effect of NETs on the host in inflammation has been studied extensively.

To understand the beneficial and harmful effect of NETs on host cells, dynamic observations of when, where, and how neutrophils release NETs is needed.

Both a spinning disk confocal microscopy [11], [13]–[15] and a multiphoton microscopy (MPM) [7], [8] have been used for *in vivo* NETs imaging, which contribute to analyze the dynamics of neutrophils at the cellular level. To explore the physiological or pathophysiological relevance of NETs, intravital imaging is necessary for direct observation of when, where, and how neutrophils release NETs.

We have developed a method of intravital imaging for intra-abdominal organs using a MPM which provides higher resolution, increased tissue penetration, and reduced photo-damage [16], [17]. The system allows us to capture high-magnification, high-resolution images of exteriorized living tissue, from the surface to several micrometers depth [18]–[23]. Previously, we have visualized in vivo real-time bacterial translocation in dextran sodium sulfate-induced colitis [18], thrombus formation in the laser-induced endothelium injury [19], three-dimensional steroid efficacy for DSS-induced colitis [20], colorectal liver metastatic formation [21], and chemotherapy response on the tumor microenvironment of colorectal liver metastases [22], [23].

In this study, we characterized NETs *in vivo* in various organs of a LPS-induced sepsis model using green fluorescent protein transgenic mice. We also investigate the associations between intravascular NETs and platelets, leukocytes, or vascular endothelium in a murine sepsis model.

## Materials and Methods

### Ethics Statement

This study was reviewed and approved by the Institutional Review Board and the Local Ethics Committee of the Mie University Graduate School of Medicine (No. 2225). Written informed consent was obtained from all the patients (adults) enrolled onto the study. The experimental protocols of in vivo studies were reviewed and approved by the Animal Care and Use Committee at the Mie University Graduate School of Medicine.

### Antibodies and Reagents

Goat anti-mouse histone H2AX and goat anti-mouse neutrophil elastase (NE) antibodies were purchased from Santa Cruz Biotechnology (Santa Cruz, CA, USA). SYTOX Green and Orange nucleic acid stains and Zenon Alexa Fluor immunoglobulin G (IgG) labeling kits were purchased from Invitrogen (Carlsbad, CA, USA). LPS (Escherichia coli, serotype 0111:B4) was purchased from Sigma-Aldrich (St. Louis, MO, USA). Deoxyribonuclease I (DNase I) was purchased from Roche Applied Science (Mannheim, Germany). Isolectin GS-IB4 conjugated with Alexa Fluor 594 was purchased from Invitrogen (Carlsbad, CA, USA). Rat anti-mouse monoclonal antibodies against Gr-1 and CD31 were purchased from BD Pharmingen (San Diego, CA, USA).

### Mice

Wild-type C57/BL6 mice and enhanced GFP (EGFP)-transgenic C57/BL6-Tg (CAG-EGFP) mice [24] were purchased from Japan SLC (Shizuoka, Japan). The 10- to 12-week-old male mice were bred, housed in groups of six mice per cage, and fed with a pelleted basal diet (CE-7; CLEA Japan, Tokyo, Japan) and had free access to drinking water. Mice were kept in the animal house facilities at the Mie University School of Medicine under standard conditions of humidity (50%±10%), temperature (23±2°C) and light (12/12-h light/dark cycle), according to the Institutional Animal Care Guidelines.

### Isolation of Human Neutrophils

Venous blood (6ml each) was obtained from healthy human volunteers (n = 5). Neutrophils were isolated by density gradient centrifugation (at 500 g for 30 min) using Polymorphprep solution (Axis Shield PoC AS, Oslo, Norway) according to the manufacturer's instructions. Neutrophils were resuspended in RPMI 1640 without phenol red supplemented with 1% fetal bovine serum. Neutrophil purity was confirmed to be routinely >90%, as assessed by May-Grünwald Giemsa staining on the blood smear. The institutional review board of the Mie University Graduate School of Medicine approved the study (No. 2225). All patients provided written informed consent for collection of samples and subsequent analysis.

### Isolation of Murine Leukocytes

Heparinized blood was withdrawn from the inferior vena cava of anesthetized wild-type C57/BL6 mice or GFP mice. Ammonium-chloride-potassium (ACK) lysing buffer (Lonza, Walkersville, MD, USA) was used to lyse the red blood cells. After ACK treatment, the blood cells that remained included white blood cells (leukocytes) and platelets. They were suspended in the above mentioned medium. Final leukocyte concentration was determined by hemacytometer. The Animal Care and Use Committee at the Mie University Graduate School of Medicine reviewed and approved the experimental protocols (No. 24–26).

### LPS-Induced Sepsis Model

LPS at a dose of 20 mg/kg was administered intraperitoneally to wild-type C57/BL6 mice or GFP mice.

### Quantification of Plasma DNA

Heparinized blood was obtained from control (LPS-untreated) and LPS-treated mice (5–10 mice at each time point). Blood plasma was separated by centrifugation and stored at −80°C until analysis. Plasma DNA was quantified using a Quant-iTTM PicoGreen dsDNA Assay Kit according to the manufacturer's instructions. In brief, plasma was diluted 10-fold with Tris-EDTA buffer and mixed with an equal volume of PicoGreen reagent. The PicoGreen dye that was bound to double-stranded DNA (dsDNA) was measured using a fluorescence microplate reader (2030 ARVO X; Perkin Elmer, Waltham, MA, USA). The DNA concentration was calculated using a standard curve generated from a series of Lambda DNA standard (100 µg/ml) provided by the manufacturer.

### Quantification of *Ex Vivo* NETs

Human neutrophils or murine leukocytes obtained from wild-type C57/BL6 mice were suspended in the above-mentioned medium. Human neutrophils or murine leukocytes (1×10^4^ per well) were seeded in 96-well plates and stimulated with LPS at indicated concentrations (2, 20, 100, and 200 µg/mL). The plates were placed in a humidified incubator at 37°C with CO2 (5%) for 6 h. To detect NETs in the culture supernatants, the culture supernatant of each well (100 µL) was collected and transferred to another well. dsDNA in the culture supernatants were measured by a PicoGreen dsDNA Assay Kit. After removal of culture supernatants, fresh medium was added to each well. The PicoGreen dsDNA Assay Kit was also used to detect NETs anchored to neutrophils on the culture well.

### Visualization of *Ex Vivo* Nets by Fluorescence Microscopy

Human neutrophils or murine leukocytes obtained from wild-type C57/BL6 mice (1×10^4^ per well) were stimulated with LPS for 6 h. A cell-impermeable DNA binding dye, SYTOX Green (5 µM), was added to each well. Ex vivo NETs stained by SYTOX Green were visualized and identified as extracellular DNAs with fibrous structures by fluorescence microscopy (IX71; Olympus, Tokyo, Japan).

### Visualization of *Ex Vivo* NETs by MPM

Human neutrophils or murine leukocytes obtained from GFP mice (5×10^5^ per well) were seeded onto 16-mm polylysine-coated coverslips in 12-well tissue culture plates and allowed to adhere at 37°C and 5% CO2 for 15 min. Then, cells were stimulated with LPS (20 µg/mL) for 6 h. Zenon Alexa Fluor IgG labeling kits were used to make Alexa Fluor 594-labeled anti-histone or anti-NE antibodies with a modified procedure [14].

For visualization of in vivo NETs by MPM, we confirmed whether SYTOX Orange, Alexa Fluor 594-labeled anti-histone, or anti-NE antibodies can interact with ex vivo NETs and whether their interactions can be visualized immediately by MPM without cell fixation (ex vivo live cell imaging).

Ex vivo NETs on coverslips were directly stained with SYTOX Orange (for human neutrophils and murine leukocytes), Alexa Fluor 594 labeled anti-histone, or anti-NE antibodies (for murine leukocytes) without cell fixation. They were visualized immediately using MPM (FV1000-2P laser-scanning microscope system; FLUOVIEW FV1000MPE, Olympus, Tokyo, Japan).

### Setup of MPM

Experiments were performed using an upright microscope (BX61WI; Olympus, Tokyo, Japan) and a FV1000-2P laser-scanning microscope system (FLUOVIEW FV1000MPE; Olympus, Tokyo, Japan) as described previously [18], [19]. The use of special stage risers enabled the unit to have an exceptionally wide working distance. This permitted the stereotactically immobilized, anesthetized mouse to be placed on the microscope stage. The microscope was fitted with several lenses with high numeric apertures to provide the long working distances required for in vivo work, and with water-immersion optics. The excitation source in MPM mode was Mai Tai Ti:sapphire lasers (Spectra Physics, Mountain View, CA), tuned and mode-locked at 910 nm. The Mai Tai produces light pulses of about 100 fs width (repetition rate 80 MHz). Laser light reached the sample through the microscope objectives, connected to an upright microscope (BX61WI; Olympus, Tokyo, Japan).

A mean laser power at the sample was between 10 and 40 mW, depending on the depth of imaging. Microscope objective lens used in this study were 20× UPlanSApo (numerical aperture 0.75) for ex vivo imaging and 60×LUMPlanFI/IR (water dipping, numerical aperture of 0.9, working distance 2 mm) for in vivo imaging, respectively. Data were analyzed using a FV10-ASW (Olympus, Tokyo, Japan). MPM images were acquired with 512×512 pixels spatial resolution, from 211-µm field of view dimension, using a pixel dwelling time 4 µs.

### Surgical Procedures for *In Vivo* MPM Imaging

As previously described [20]–[23], GFP mice were anesthetized with isofluorane 4 L/min (4%; Forane, Abbott, Japan). Body temperature was kept at 37°C using a heating pad. For imaging postcapillary venules, the cecum was identified and exteriorized through a laparotomy. The exteriorized cecum was optimally inflated by introducing air using a syringe with a small-bore needle. The inflated cecum was placed on an organ-stabilizing system (Japanese Patent No. 5268282) to reduce the effect of heartbeats and respiratory movements. For imaging hepatic sinusoids, the left lateral lobe of the liver was identified and exteriorized through a laparotomy. The liver lobe was put on an organ-stabilizing system using a solder lug terminal with an instant adhesive agent.

### Imaging Methods

The surface of the cecum or liver was initially screened at lower magnifications by setting out the X/Y plane and adjusting the Z axis manually to detect the optimal observation area [19]–[22]. Each area of interest was subsequently scanned at a higher magnification (water-immersion objective 60× with or without 2× zoom) by manually setting the X/Y plane and adjusting the Z axis (either automatically or manually) to obtain high-resolution, clear MPM images. The scanning areas were 211×211 µm (600×) or 106×106 µm (600× with 2×zoom) respectively. The imaging depth was determined as a length from the tissue surface. In our experimental setting, the imaging depth ranged from 100 µm to 400 µm. The optimal high resolution images were obtained from the tissue surface up to 100 to 200 µm depth. The laser power was adjusted according to the imaging depth. When imaging at larger depths, we increase the laser power level (up to 100%) manually using laser power level controller. To image the optimal simultaneous imaging of EGFP and Alexa Fluor 594, detection sensitivity (brightness by HV) was adjusted manually for EGFP (485–510) or Alexa Fluor 594 (to 585–635), respectively.

### Visualization of *In Vivo* NETs by MPM

LPS (20 mg/kg i.p.) was administered to GFP mice. After 12–24 h, systemic clinical signs such as reduced motor activity, lethargy, shivering, and piloerection were observed. The mice with these signs were used as LPS treated mice. To administer regents precisely and reliably, a catheter (M-FAC/FVC, Neuroscience, Tokyo, Japan) was placed in the femoral vein of the anaesthetized mice under surgical microscopy. After laparotomy, the cecum or liver was exteriorized and fixed using the organ-stabilizing system.

After identifying optimal areas, SYTOX Orange, Alexa Fluor 594-labeled anti-histone, or anti-NE antibodies were administered intravenously via a catheter. NETs were detected and visualized in the living mice. Color-coded green (EGFP) and red (SYTOX Orange and Alexa Fluor 594) images were recorded at the same time. Subsequently, they were merged to produce single dual-color images.

Alexa Fluor 594-labeled mouse IgG was used as isotype control for either the LPS-treated or normal control mice.

### DNase I Treatment for *In Vivo* NETs

DNase I treatment has been used for the confirmation of the presence of NETs either in vitro or in vivo, since NETs (extracellular DNAs) were degraded or subtracted by DNase I [11], [13]–[15].

After the identification of in vivo NETs in postcapillary venules of the cecum of LPS treated mice, DNase I at a dose of 1000 U was administered intravenously via a catheter (DNase I treatment group, n = 5). In control group (n = 5), the equal amount of phosphate buffered saline (PBS) was administered after NETs identification.

To minimize the photobleaching for NETs during the observation, we recorded the time course of change in NETs by a method of 30 sec-imaging followed by 90 sec-pause for at least 30 min (at least 15 cycles) manually.

### Quantification of Leukocyte-Endothelial Interaction *In Vivo*

We observed that leukocytes were adhering to the vascular endothelium of the postcapillary venules in LPS-injected mice.

The number of leukocyte-endothelial interactions was counted in 10 randomly selected fields (×600; 211 µm×211 µm square) with intravital MPM imaging.

The average number per field (211 µm×211 µm square) was expressed as the number of leukocyte-endothelial interaction per field of view (FOV).

### Quantification of *In Vivo* NETs

Intravascular NETs were identified by SYTOX Orange, Alexa Fluor 594-labeled anti-histone antibody, or Alexa Fluor 594-labeled anti-NE antibody.

The number of NETs present in intravascular spaces, hepatic sinusoids, and pulmonary capillaries was counted in 10 randomly selected fields (×600; 211 µm×211 µm square) with intravital MPM imaging. The sum of them (a total number of 10 fields) was expressed as the number of NETs per field of view (FOV).

### Quantification of Endothelial Injury Using Isolectin GS-IB4

To visualize vascular endothelium in vivo real-time, isolectin GS-IB4 conjugated with Alexa Fluor 594 (invitrogen, Carlsbad, CA, USA) were administered to the living mice [19] Isolectin GS-IB4 positive endothelial cells were visualized in hepatic sinusoids (in vivo) or pulmonary capillaries (ex vivo). Endothelial injury was defined as isolectin GS-IB4 negative microvascular area.

Isolectin GS-IB4 positive endothelial cells were evaluated in 10 randomly selected fields (×600; 211 µm×211 µm square) by MPM imaging. The percentage of endothelial integrity (isolectin GS-IB4 positive staining) was calculated by dividing the area of isolectin GS-IB4 positive staining by the total area of microvascular area.

Finally, endothelial integrity was scored as 0 (0%), 1 (1–25%), 2 (26–50%), 3 (51–75%), and 4 (76–100%) according to the percentage of isolectin GS-IB4 positive endothelial cells. The endothelial integrity per FOV (211 µm×211 µm square) was determined by averaging the scores of 10 FOVs.

### Measurement of Blood Flow Speed

Blood flow speed was evaluated using the line-shift-diagram method in a 100-µm segment of the vessel [19]. In brief, platelet velocity was calculated by measuring the distance of platelet traveled between two consecutive images divided by the time interval between these two images. The platelet for blood velocity measurement was carefully selected after confirming to be identical between two consecutive images.

### Immunohistochemistry for Gr-1 and CD31

Tissue sections (7 µm thick) were washed in phosphate-buffered saline (PBS), and immersed in a 0.3% hydrogen peroxide in methanol for 5 min to block endogenous peroxidase activity. Nonspecific-binding sites were blocked with normal goat serum (Vector Laboratories Inc., Burlingame, CA, USA) for 60 min. Sections were then incubated with primary rat anti-mouse monoclonal antibodies against Gr-1 (1∶250; BD Pharmingen, San Diego, CA, USA) and CD31 (1∶100; BD Pharmingen, San Diego, CA, USA) for 2 h at 4°C. After washing with PBS, sections were incubated with a horseradish peroxidase (HRP)-conjugated goat anti-mouse IgG (H+L) (KPL, Gaithersburg, MD, USA) at a 1∶200 dilution for 30 min at room temperature. The specific staining was visualized via a 3,3′-diaminobenzidine (DAB) substrate. The sections were counterstained with Mayer’s hematoxylin. As negative control, tissue sections were prepared by omitting the primary antibody.

### Quantification of Neutrophil Recruitment By Gr-1 Immunostaining

Neutrophils were identified as Gr-1 immunoreactive cells. Gr-1 positive neutrophils were counted in a high-power field (HPF; ×200) of 10 randomly selected areas by a light microscopy. The number of neutrophil recruitment per HPF was determined by averaging the counts of 10 HPFs.

### Quantification of Endothelial Injury by CD31 Immunostaining

Endothelial cells were quantified by CD31 immunostaining. CD31 positive staining was detected in hepatic sinusoids or pulmonary capillaries. Endothelial injury was defined as CD31 negative microvascular area.

CD31 immunostaining was evaluated in a high-power field (HPF; ×200) of 10 randomly selected areas by a light microscopy. The percentage of endothelial integrity (CD31 positive staining) was calculated by dividing the area of CD31 positive staining by the total area of microvascular area.

Finally, endothelial integrity was scored as 0 (0%), 1 (1–25%), 2 (26–50%), 3 (51–75%), and 4 (76–100%) according to the percentage of CD31 positive endothelial cells.

The endothelial integrity per HPF was determined by averaging the scores of 10 HPFs.

### Statistical Analysis

Statistical analyses were performed using JMP version 5 (SAS Institute Inc. Cary, NC, USA). The data were presented as the mean+standard error (SE) and were analyzed by Mann-Whitney U test. P value of less than 0.05 was considered statistically significant.

## Results

### Cell-Free and Anchored Nets *In Vitro*

NETs have two distinct forms [11], [13]–[15]. One form is NETs that are released away from neutrophils (cell-free NETs), and the other is those that are anchored to neutrophils (anchored NETs). We examined the LPS induced cell-free and anchored NETs in vitro.

SYTOX Green detected NETs was significantly higher in culture supernatants than that of control in an LPS dose-dependent manner (Figure S1-A). Anchored NETs on culture wells after removal of culture supernatants was significantly higher than that of control wells in an LPS dose-dependent manner (Figure S1-B). Fluorescence microscopy showed that SYTOX Green stained both extracellular DNAs that were anchored to neutrophils (anchored NETs) and nuclei of non-viable neutrophils (Figure S1-C).

### Morphological Characteristics of Nets *Ex Vivo*

We examined morphological features of NETs ex vivo. NETs were stained by SYTOX Orange (A), Alexa Fluor 594-labeled anti-histone antibody (B), and Alexa Fluor 594-labeled anti-NE antibody (C).

By SYTOX Orange staining, NETs were characterized as linear structures (Figure S2-A-i), reticular structures anchored to leukocytes (Figure S2-A-ii), reticulolinear structures anchored to leukocytes (Figure S1-A-iii), and spot-like structures anchored to leukocytes (Figure S2-A-iv).

Morphological characteristics of NETs stained by Alexa Fluor 594-labeled anti-histone (Figure S2-B) or NE (Figure S2-C) antibody were similar to those stained by SYTOX Orange. NETs were also characterized as membranous structures (Figure S2-C-iii).

### Plasma Cell-Free DNA in a Murine Sepsis Model

Plasma cell-free DNA has been used as a surrogate marker of circulating cell-free NETs [5], [6]. To determine the optimal timing of the observation of in vivo NETs, we examined the amount of plasma DNA at the indicated time points after LPS administration.

As shown in Figure S3, plasma DNA was significantly increased in LPS-treated mice compared with normal control mice with a peak at 24 h after the intraperitoneal administration of LPS at a dose of 20 mg/kg whose mortality rate was approximately 50%.

### *In Vivo* Nets in Postcapillary Venules of the Cecum

The leukocyte-endothelial interaction was frequently initiated at the postcapillary venules [25]. Since the LPS-induced adhesion of leukocytes or platelet binding leukocytes to the vascular endothelium was observed in postcapillary venules of the cecum, we hypothesized that anchored NETs might be observed there most frequently.

**Figure 1:**
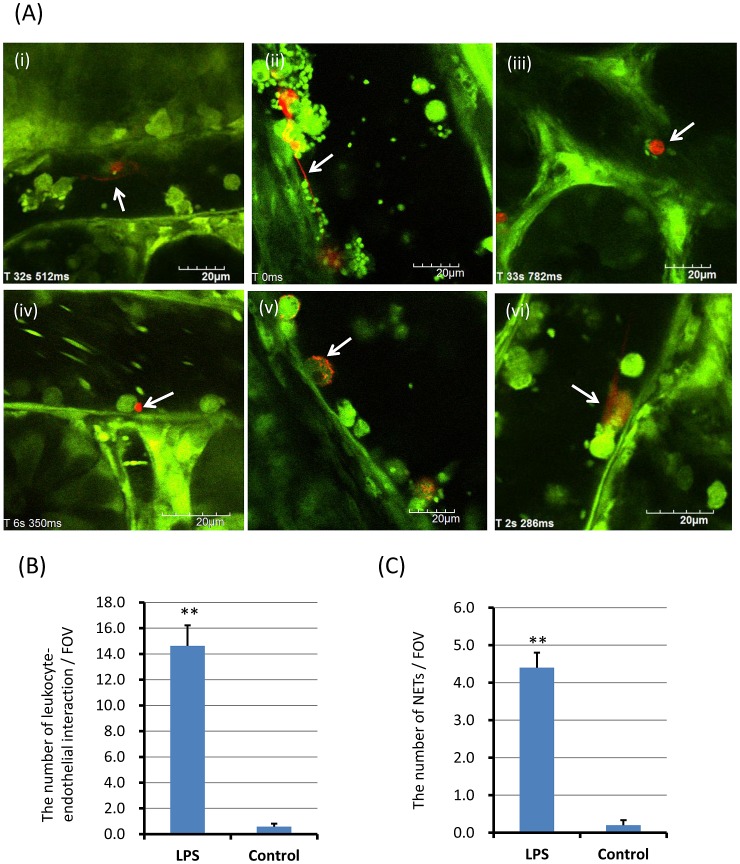
*In vivo* NETs in postcapillary venules of the cecum. The postcapillary venules of the cecum were observed at 24 h after LPS (20 mg/kg) intraperitoneal administration (n = 10). NETs (red) were detected by SYTOX Orange, Alexa Fluor 594-labeled anti-histone antibody, or Alexa Fluor 594-labeled anti-NE antibody, respectively. NETs were characterized as reticular structures anchored to leukocytes (A-i; Movie S1), reticulolinear structures (A-ii), spot-like structures anchored to leukocytes (A-iii, iv), membranous structures on the surface of leukocytes (A-v) and linear structures anchored to leukocytes (A-vi; Movie S2). The number of leukocyte-endothelial interactions and NETs per FOV was determined as described in materials and methods, respectively. The number of leukocyte-endothelial interactions per FOV (B) were significantly greater in LPS treated mice than control mice (14.6±1.6 vs 0.6±0.2). The number of NETs per FOV (C) was also significantly greater in LPS treated mice than control mice (4.4±0.4 vs 0.2±0.1). Data was presented as mean+standard error. **P<0.01 versus control.

After the intraperitoneal administration of LPS at a dose of 20 mg/kg, NETs were observed as reticular structures anchored to leukocytes (Figure 1-A-i; Movie S1), reticulolinear structures (Figure 1-A-ii), spot-like structures anchored to leukocytes (Figure 1-A-iii, iv), membranous structures on the surface of leukocytes (Figure 1-A-v) and linear structures anchored to leukocytes (Figure 1-A-vi; Movie S2). Neither anchored nor circulating cell-free NETs were observed in normal control mice. In addition, no rolling or circulating leukocytes with NETs were seen.

The number of leukocyte-endothelial interactions per FOV and the number of NETs per FOV were determined as described in materials and methods. The number of leukocyte-endothelial interactions per FOV were significantly greater in LPS treated mice than control mice (Fig. 1-B; 14.6±1.6 vs 0.6±0.2, p<0.01). The number of in vivo NETs per FOV was also significantly greater in LPS treated mice than control mice (Fig. 1-C; 4.4±0.4 vs 0.2±0.1, p<0.01). The percentage of NETs to leukocyte-endothelial interactions was approximately 3% (4.4/146). NETs seemed to be the rare event in vivo, as compared with leukocyte-endothelial interaction.

Interestingly, we observed leukocytes showing cytoplasmic vacuoles that adhered to vascular endothelium in LPS-treated mice at the subcellular level (Figure S4-i, ii; arrows). They were more frequently observed in postcapillary venules of the cecum than arterioles or hepatic sinusoids. Some of them released NETs (Figure S4-iii, iv; arrows). These findings may indicate a NETosis, which is defined as a distinct cell death related to NETs [26], [27].

### *In Vivo* NETs in Hepatic Sinusoids of the Liver

**Figure 2:**
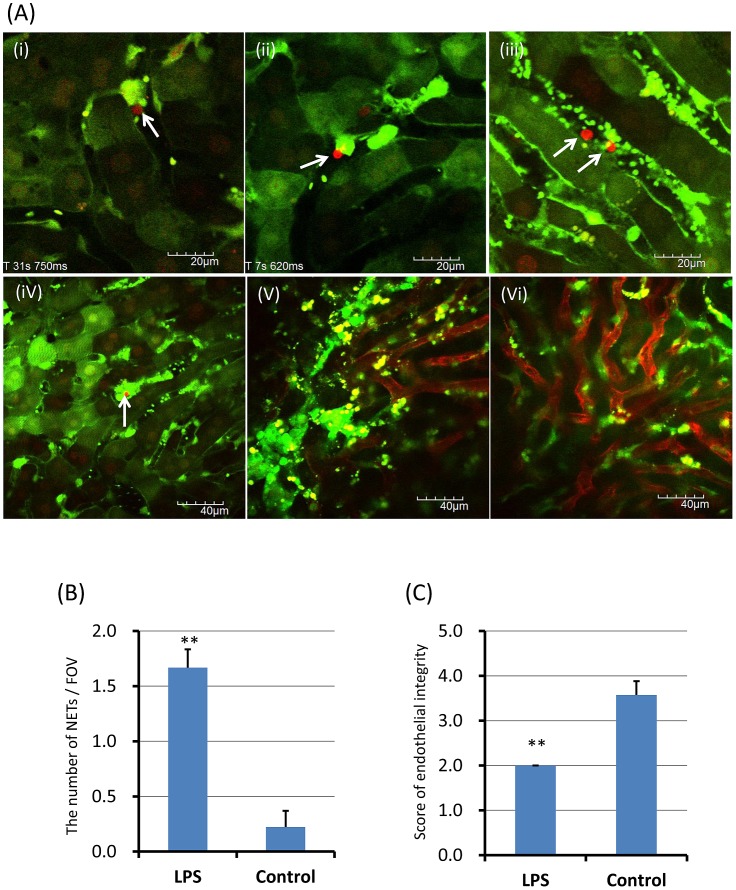
*In vivo* NETs in hepatic sinusoids of the liver. The hepatic sinusoids were observed at 24 h after LPS (20 mg/kg) intraperitoneal administration (n = 10). NETs (red) were detected by SYTOX Orange, Alexa Fluor 594-labeled anti-histone antibody, or Alexa Fluor 594-labeled anti-NE antibody, respectively. NETs were characterized as spot-like structures anchored to leukocytes (A-i, ii; Movie S3), cell-free DNA fragments (A-iii; Movie S4), and cell-free DNA fragments within platelet aggregates (A-iv). Isolectin GS-IB4 stained hepatic sinusoidal endothelial cells were observed in LPS-treated mice (A-v) and normal mice (A-vi). The number of NETs per FOV and the score of endothelial integrity were determined as described in materials and methods, respectively. The number of NETs per FOV (B) was significantly greater in LPS treated mice than normal control mice (1.7±0.2 vs 0.2±0.1). The score of endothelial integrity (C) was significantly less in LPS treated mice than control mice (.0±0.3 vs 3.6±0.2). Data was presented as mean+standard error. **P<0.01 versus control.

Neutrophils recruit to hepatic sinusoids during inflammation [11], [13], [14]. After LPS administration, leukocyte accumulation and platelet aggregation were observed in hepatic sinusoids of the liver. NETs were also observed as spot-like structures anchored to leukocytes (Figure 2-A-i, ii; Movie S3), cell-free DNA fragments (Figure 2-A-iii; Movie S4), and cell-free DNA fragments within platelet aggregates (Figure 2-A-iv). The number of in vivo NETs per FOV was significantly greater in LPS treated mice than normal control mice (Figure 2-B; 1.7±0.2 vs 0.2±0.1, p<0.01).

NETs have been reported to cause endothelial dysfunction or injury [27], [28]. To visualize endothelial injury of hepatic sinusoids, isolectin GS-IB4 conjugated with Alexa Fluor 594 was used as described in materials and methods. Isolectin GS-IB4 stained hepatic sinusoidal endothelial cells were observed in LPS-treated mice (Figure 2-A-v) and normal mice (Figure 2-A-vi). The score of endothelial integrity based on the percentage of isolectin GS-IB4 positive endothelial cells was significantly lower in LPS treated mice than control mice (Figure 2-C; 2.0±0.3 vs 3.6±0.2, p<0.01).

Contrary to previous reports [11], [13], widespread web-like NETs was not observed in hepatic sinusoids. Therefore, the endothelial injury in hepatic sinusoids might be mainly caused by severe LPS-induced inflammation rather than NETs.

### NETs in Pulmonary Capillaries of the Lung

After imaging of the cecum and liver, excised lungs (ex vivo) were observed immediately to examine NETs in the lung.

**Figure 3:**
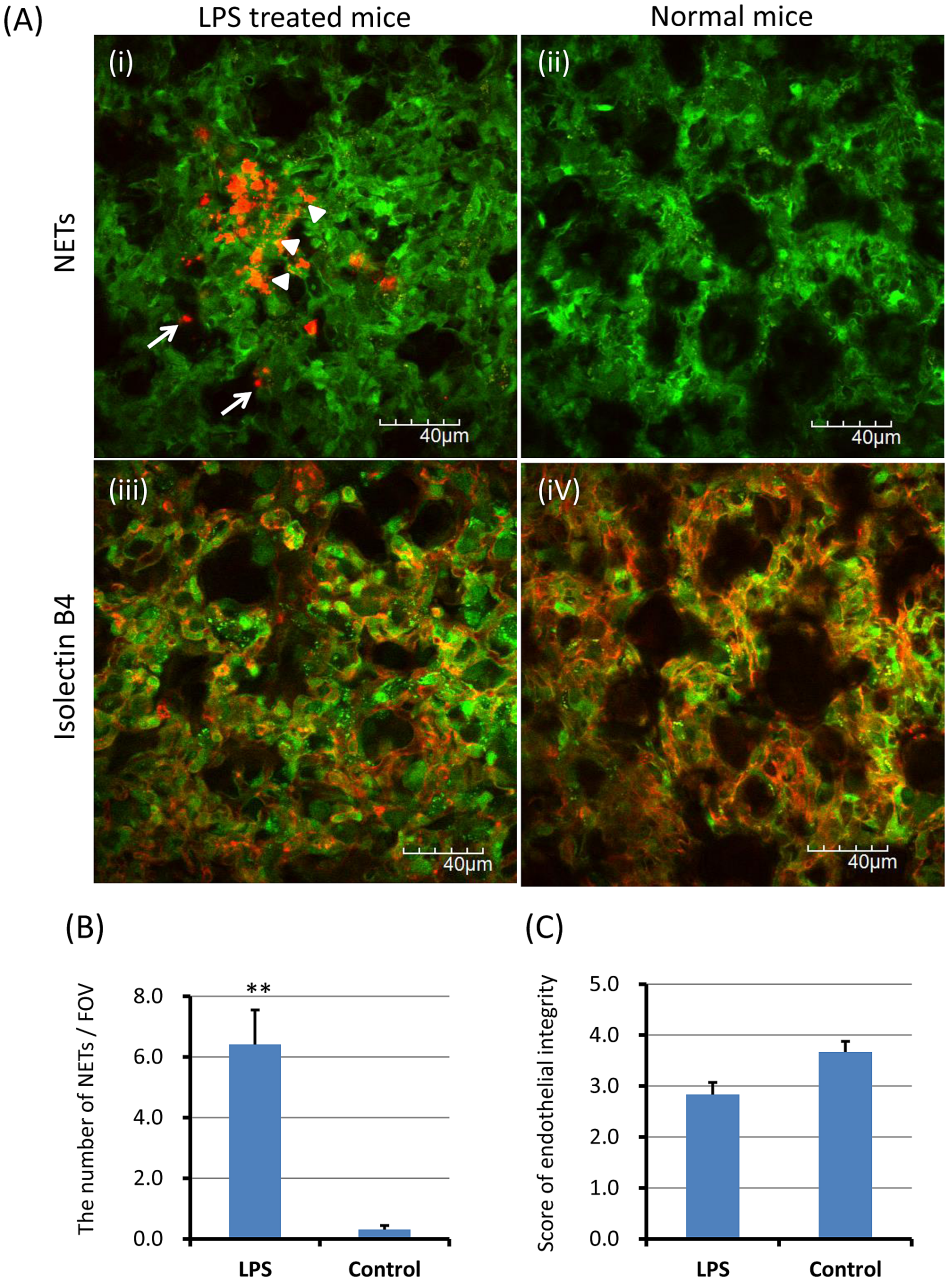
NETs in pulmonary capillaries of the lung. The excised lungs were investigated immediately after intravital imaging of the cecum and liver to evaluate NETs and endothelial injury. NETs (red) were observed in alveolar space (A-i; arrows) and pulmonary capillaries (A-i; arrowheads) of LPS-treated mice. The number of NETs per FOV and the score of endothelial integrity were determined as described in materials and methods, respectively. The number of NETs per FOV was significantly greater in LPS-treated mice than control mice (B; 6.4±1.1 vs 0.3±0.1). The score of endothelial integrity (C) was lower in LPS treated mice than control mice (2.8±0.2 vs 3.7±0.2). Data was presented as mean+standard error. **P<0.01 versus control.

NETs were observed in alveolar space (Figure 3-A-i; arrows) and pulmonary capillaries (Figure 3-A-i; arrowheads) of LPS-treated mice. The number of NETs per FOV was significantly greater in LPS-treated mice than control mice (Figure 3-B; 6.4±1.1 vs 0.3±0.1, p<0.01). Circulating cell-free NETs appeared to be entrapped in pulmonary capillaries rather than hepatic sinusoids.

Isolectin GS-IB4 stained alveolar capillary endothelial cells of excised lungs were observed in LPS-treated mice (Figure 3-A-iii) and normal mice (Figure 3-A-iv). The score of endothelial integrity based on the percentage of isolectin GS-IB4 positive endothelial cells was 2.8±0.2 in LPS treated mice and 3.7±0.2 in normal control mice, respectively. The difference was not statistically significant (Figure 3-C; p = 0.057).

Contrary to the previous reports [11], [14], widespread web-like NETs was not observed in pulmonary capillaries, indicating that the endothelial injury might be mainly caused by LPS-induced inflammation rather than NETs.

### Circulating Cell-Free NETs

**Figure 4:**
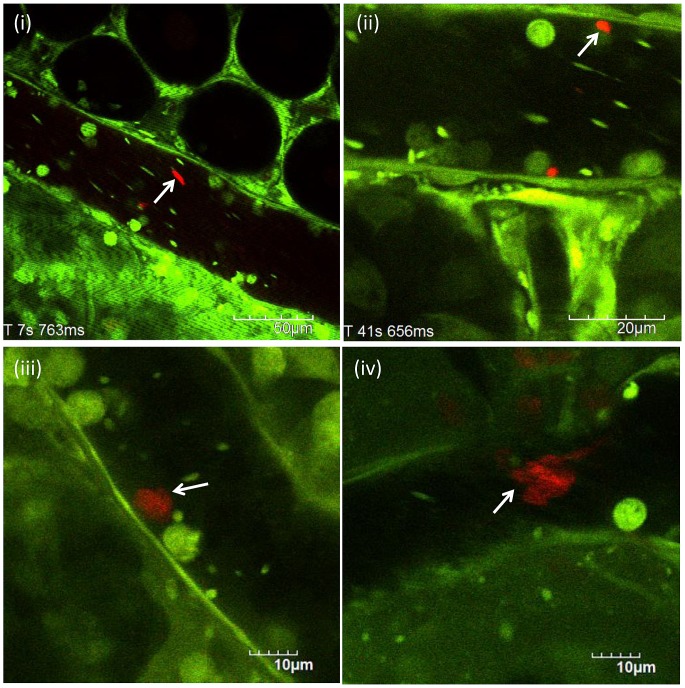
Circulating cell-free NETs. Circulating cell-free NETs (red) were observed in the blood flow of either postcapillary venules of the cesum or hepatic sinusoids of the liver (i, ii) at 24 h after LPS (20 mg/kg) intraperitoneal administration (n = 10). NETs were anchored to the leukocyte adhering to the vascular endothelium (iii; Movie S5). Thereafter, NETs were leaving away from leukocyte, changing shape from solid to cotton-like, and flowing down the vessel. These cotton-like NETs were arrested far away from the leukocyte (iv). They were not observed in control mice.

Circulating cell-free NETs were observed in the blood flow of postcapillary venules of the cecum (Figure 4-i, ii) as well as hepatic sinusoids of the liver (Figure 2-A-iii, iv). Circulating cell-free NETs were characterized as fragmented or cotton-like structures. Their shape seemed to depend on blood flow speed.

Figure 4-iii (Movie S5) shows that NETs were anchored to the leukocyte adhering to the vascular endothelium. Thereafter, NETs were leaving away from leukocyte, changing shape from solid to cotton-like, and flowing down the vessel. These cotton-like NETs were arrested far away from the leukocyte (Figure 4-iv).

### Association between *In Vivo* Nets and Platelets, Endothelial Cells, or Leukocyte-Platelet Aggregates

NETs have been reported to induce platelet aggregation followed by thrombus formation [5], and also induce endothelial damage or dysfunction [28], [29]. We observed the association between in vivo NETs and platelets, leukocytes, or vascular endothelium.

**Figure 5:**
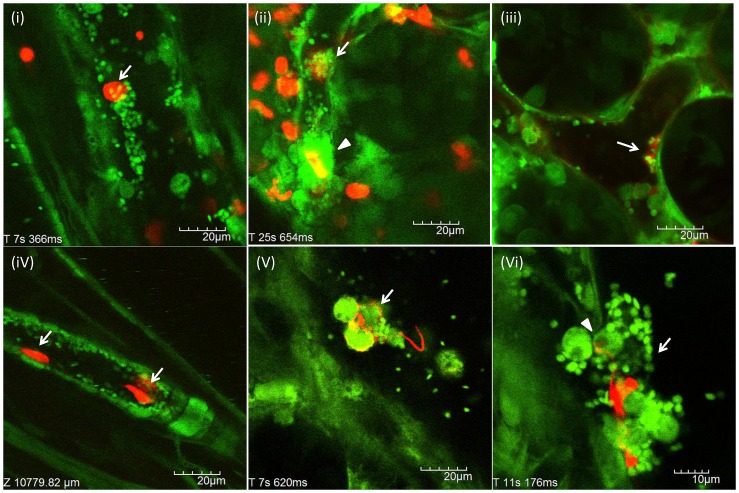
Association between in vivo NETs and platelets, endothelial cells, or leukocyte-platelet aggregates. Interactions of circulating cell-free NETs or anchored NETs with platelets, leukocytes, or vascular endothelium were investigated (n = 10, LPS treated mice). Circulating cell-free NETs (red) were binding to platelet aggregates (i; Movie S6). Platelets were aggregating to cell-free NETs (ii; arrow). Platelet thrombus formation was observed, in which cell-free NETs appeared to be a template for thrombus (ii; arrowhead). Circulating cell-free NETs were also adhering to vascular endothelium in both venule (iii) and arteriole (iv; Movie S7). NETs-leukocyte-platelet aggregates were observed in venules (v, vi; arrow). NETs seemed to be released from the leukocyte with cytoplasmic vacuoles (vi; arrowhead, Movie S8).

Circulating cell-free NETs were binding to platelet aggregates (Figure 5-i; Movie S6). Platelets were aggregating to cell-free NETs (Figure 5-ii; arrow). Furthermore, cell-free NETs appeared to be a template for platelet thrombus formation (Figure 5-ii; arrowhead). Circulating cell-free NETs were also adhering to vascular endothelium in both venule (Figure 5-iii) and arteriole (Figure 5-iv; Movie S7). NETs-leukocyte-platelet aggregates were observed in venules (Figure 5-v, vi). NETs seemed to be released from the leukocyte with cytoplasmic vacuoles (Figure 5-vi; arrowhead, arrow, Movie S8) [25], [26].

These observations may suggest that cell-free NETs seem to interact with platelets, leukocytes, or vascular endothelium.

Although the direct effect of NETs on these phenomena could not yet be clarified in this study, our observational findings may support the previous reports about the association between NETs and thrombus formation or endothelial cell damage [5], [28], [29].

### Association between *In Vivo* Nets and Microcirculation

Microvascular thrombosis may cause microvascular occlusions, resulting in tissue ischemia and organ failure [30], [31].

**Figure 6:**
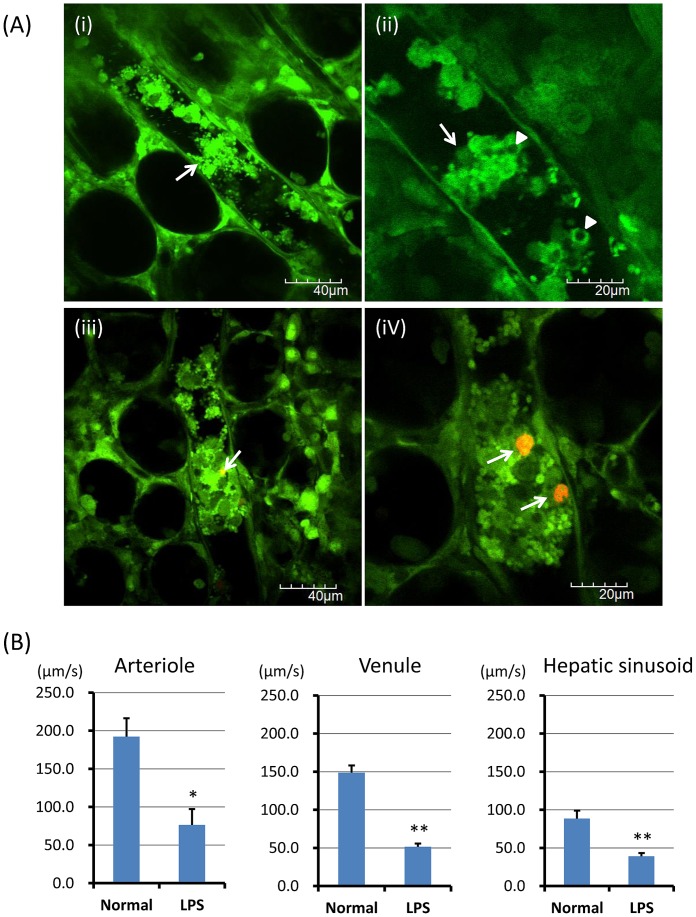
Association between in vivo NETs and microcirculation. Approximately 50% of mice were died from a lethal dose of LPS administration. The survived mice were used as LPS treated mice. In the almost dying mice (n = 10) among the LPS treated mice, microvessels were frequently occluded by platelet aggregates or leukocyte-platelet aggregates (A-i, ii; arrows, Movie S9). In some of them, cell-free NETs (red) appeared to be a template for leukocyte-platelet aggregates (A-iii, iv; arrows). Leukocyte-platelet aggregates sometimes included leukocytes with cytoplasmic vacuoles (A-ii; arrowheads). Blood flow speeds of arteriole, venule, and hepatic sinusoid (B) were significantly lower in LPS-treated mice than control, respectively. Data was presented as mean+standard error. *P<0.05, and **P<0.01 versus control, respectively.

Since the mortality rate at 24 h after the intraperitoneal administration of LPS at a dose of 20 mg/kg was approximately 50%, the survived mice (the other 50% of LPS administered mice) were used as LPS treated mice in this study. We observed that the microvessel appeared to be occluded by platelet aggregates or leukocyte-platelet aggregates in the almost dying mice. (Figure 6-A-i, ii; arrows, Movie S9). In some of them, cell-free NETs appeared to be a template for leukocyte-platelet aggregates (Figure 6-A-iii, iv; arrows). Leukocyte-platelet aggregates sometimes included leukocytes with cytoplasmic vacuoles (Figure 6-A-ii; arrowheads).

The blood flow of cecal venules and hepatic sinusoids in LPS-treated mice showed a to-and-fro movement and a heterogeneous flow by location. Furthermore, the blood flow of cecal venules and hepatic sinusoids was impaired earlier than that of arterioles (data not shown). As shown in Figure 6-B, blood flow speeds of arteriole (p<0.05), venule (p<0.01), and hepatic sinusoid (p<0.01) were significantly lower in LPS-treated mice than control, respectively.

The decreased blood flow in microcirculation may be in part caused by heterogeneous microvessel occlusion by platelet aggregates or leukocyte-platelet aggregates. Although several factors are involved in microcirculatory disturbance of a sepsis, the microvessel occlusions with platelet aggregates or leukocyte-platelet aggregates may be also one of the causes of microcirculatory disturbance in a sepsis. In addition, cell-free NETs appeared to be associated with the formation of platelet aggregates or leukocyte-platelet aggregates. However, our observational findings are still insufficient to prove the effect of NETs on microcirculatory disturbances.

### Time Course of Change in Nets by Dnase I Treatment *In Vivo*

**Figure 7:**
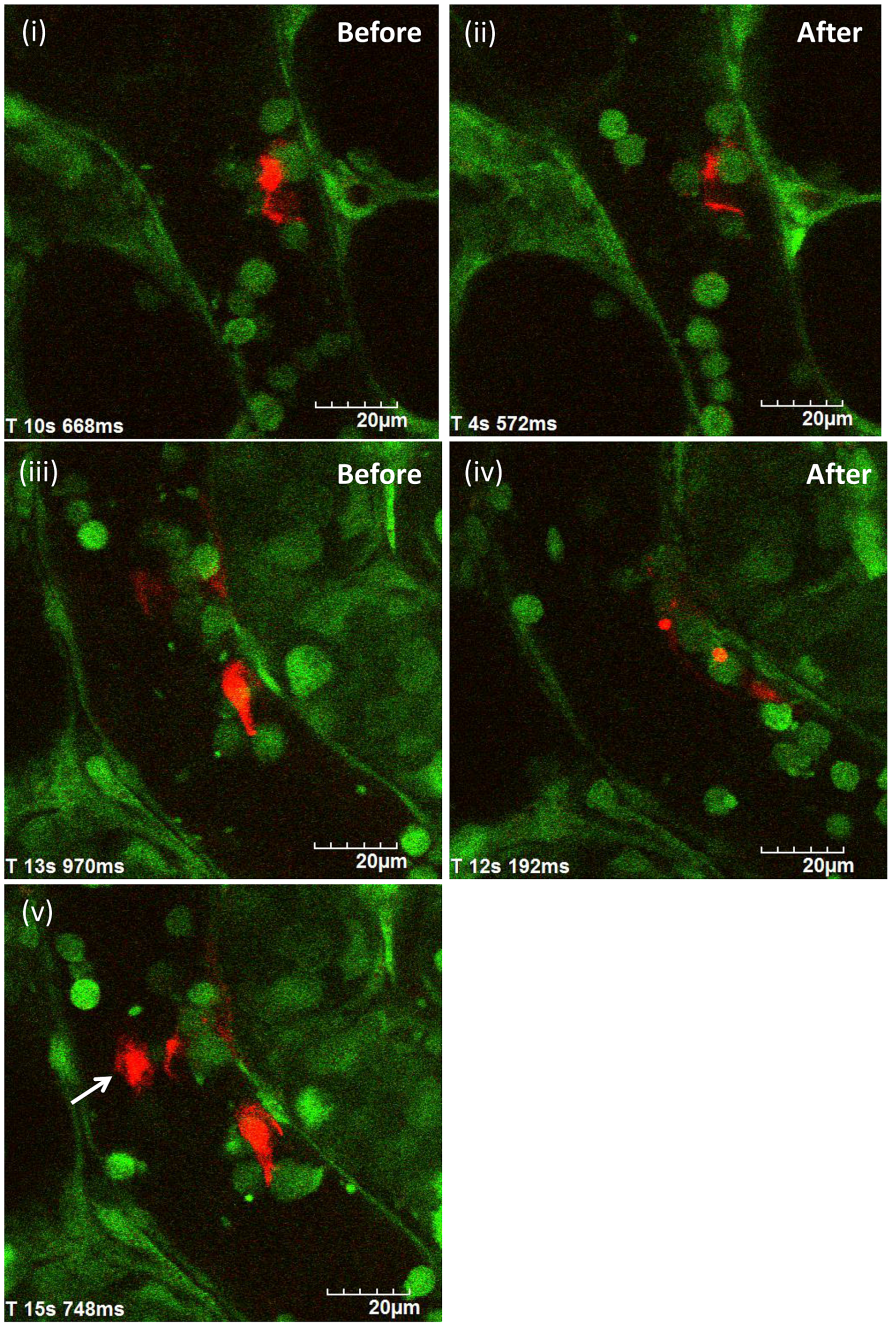
Time course of change in NETs by DNase I treatment *in vivo*. After the identification of in vivo NETs (red) in postcapillary venules of the cecum of LPS treated mice, DNase I at a dose of 1000 U was administered intravenously via a catheter (DNase I treatment group, n = 5). In control group (n = 5), the equal amount of phosphate buffered saline (PBS) was administered after NETs identification. Images were recorded by a method of 30 sec-imaging followed by 90 sec-pause for at least 30 min (at least 15 cycles) to minimize the photobleaching for NETs during the observation. Anchored NETs with optimal size in postcapillary venules of the cecum were selected to examine the time course of change in NETs by DNase I treatment. Figure 7 shows in vivo NETs before (Figure 7-i, iii) and after (Figure 7-ii, iv, v) DNase I treatment.

To examine the time course of change in NETs by DNase I treatment, anchored NETs with optimal size were selected in postcapillary venules of the cecum. Figure 7 shows in vivo NETs before (Figure 7-i, iii) and after (Figure 7-ii, iv) DNase I treatment.

As shown Figure 7-i, ii, NETs were degraded by DNase I treatment with decrease in both its shape and fluorescent intensity within 30 min. Figure 7-iii, iv showed that anchored NETs were disappeared nearly completely without falling apart from neutrophils. No more change in both shape and fluorescent intensity of NETs was found over 30 min in our experimental conditions.

In some cases, massive cell-free NETs were observed in the blood flow after DNase I injection (Figure 7-v; arrow). Since such massive cell-free NETs were not observed in non-DNase I treatment group, it was speculated that the upstream anchored NETs may fall apart from neutrophils and may subsequently circulate in the blood.

There was no significant change in both shape and fluorescent intensity of NETs by the injection of the equal amount of PBS (data not shown).

### Neutrophil Recruitment and Endothelial Injury in LPS Treated Mice

We also evaluated neutrophil infiltration and endothelial injury in the liver and lung of LPS treated mice by immunohistochemistry as a conventional morphological analysis.

As shown in Figure S5, the number of Gr-1 positive neutrophils was significantly higher in both liver (p<0.01) and lung (p<0.01) of LPS-treated mice than normal control mice, respectively. The score of endothelial integrity based on the percentage of CD31 positive endothelial cells was significantly lower in the lung (p<0.05) than normal control mice. The score of endothelial integrity was lower in the liver than normal control mice, but the difference was not statistically significant (p = 0.057).

## Discussion

In this study, two forms of NETs were clearly characterized *in vivo*: one form is cell-freeNETs that are released away from neutrophils and circulating in the bloodstream, while the other is anchored NETs that are anchored to neutrophils adhering to the vascular endothelium.

Morphologically, anchored NETs were characterized as linear, reticular, membranous, or spot-like structures. In contrast, cell-free NETs were also characterized as fragmented or cotton-like structures. The association between these morphological features and LPS-induced inflammatory response could not be clarified, because the detection rate of anchored NETs was extremely low as compared with that of leukocyte-endothelial interactions (approximately 3%). Some reports demonstrated that the frequency of NETs might be fairly low even under optimal conditions in vitro. Among one-third of activated neutrophils, less than 10% of them might release NETs [32]. Our results may be consistent with these findings.

In vivo NETs have been characterized morphologically and functionally as previously reported [11], [13]–[15]. McDonald et al demonstrated the LPS induced neutrophil recruitment to the liver and subsequent the production of intravascular NETs (hepatic sinusoids) [13]. They observed that NETs formed large extracellular webs which covered hepatic sinusoids far distant from the nearest neutrophil. Clark et al showed NETs within pulmonary capillaries of an LPS treated mouse [14]. Cools-Lartigue et al also revealed the widespread deposition of NETs within hepatic and pulmonary microvasculature of mice treated with cecal ligation and puncture (CLP) [11]. These all reports show the induction of widespread web-like NETs expanding the entire hepatic sinusoids and pulmonary capillaries by LPS injection or CLP.

Since NETs have been reported to be formed from an adherent neutrophil in vitro [14], we firstly observed postcapillary venules where leukocyte-endothelial interactions were frequently initiated and observed to detect in vivo NETs effectively. Unexpectedly, a few NETs (either cell-free or anchored form) were observed in postcapillary venules of the cecum. Contrary to the findings of in vivo NETs in previous reports [11], [13], [14], a few NETs were observed in both hepatic sinusoids and pulmonary capillaries.

We didn’t observed neither the excess production of NETs after excess neutrophil recruitment nor the accumulation of circulating cell-free NETs in relatively low shear vascular bed such as hepatic sinusoids and pulmonary capillaries. The difference in findings of in vivo NETs between ours and the others [11], [13], [14] might partly depend on LPS dosage, timing of imaging, and detection method.

Recently, NETs research has focused on its adverse effect on the host [5]–[12] as well as the distinct function of extracellular trapping and killing pathogens [3], [4]. Both nuclear and granular proteins contained in NETs seemed to induce NETs-mediated host cell cytotoxicity [28], [29].

In this study, cell-free NETs appeared to interact with platelets, leukocytes, or vascular endothelium of arterioles and venules in the microcirculation of a murine sepsis model. Although we could not yet clarify the direct effect of NETs on these phenomena, our observational findings may support the previous reports about the association between NETs and thrombus formation or endothelial cell damage [5], [28], [29].

Sepsis is still a life-threatening condition, and defined as a systemic response to infection with the presence of organ dysfunction or failure [30], [31].

The almost dying septic mice showed either complete or incomplete microvessel occlusions, which may seem to be caused by platelet aggregates or leukocyte-platelet aggregates. Although several factors are involved in microcirculatory disturbance of a sepsis, the microvessel occlusions with platelet aggregates or leukocyte-platelet aggregates may be also one of the causes of microcirculatory disturbance in a sepsis.

Our observational findings are still insufficient to prove the effect of NETs on microcirculatory disturbances of a sepsis. However, the findings that cell-free NETs appeared to be a template for platelet aggregates or leukocyte-platelet aggregates may suggest the adverse effect of NETs on the host in a sepsis.

We also observed leukocytes showing cytoplasmic vacuoles. Most of them were adhering to the vascular endothelium of postcapillary venules. These leukocytes were considered as NETosis [26], [27]. Although we didn’t examine the relationship between NETosis and anchored NETs or cell-free NETs, the number of NETosis (leukocytes showing cytoplasmic vacuoles) seemed to be greater than that of anchored NETs (leukocytes attaching NETs). This might be consistent with the results of quantification of cell-free and anchored NETs in vitro.

There are several limitations in our current setting, such as a limited observation area and a limited imaging time [18]–[23]. The number of interactions of in vivo NETs with platelet, leukocytes, and vascular endothelium observed in our experimental conditions is extremely low even in lethal condition. To proceed the research of NETs-host interactions, it is necessary to modify our experimental conditions.

However, this is the first report that has presented morphological characterization of in vivo NETs and the interactions of in vivo NETs with platelet aggregates, leukocyte-platelet aggregates and vascular endothelium in the microcirculation of a murine sepsis model.

Although further study is needed to clarify the direct effect of NETs on these observations, our results might be useful for the future NETs research.

## Supporting Information

Figure S1 **Cell-free and anchored NETs** ***in vitro*** **.** NETs were classified as two forms: NETs that are released away from neutrophils (cell-free NETs), and those that are anchored to neutrophils (anchored NETs). In vitro study, extracellular DNA in culture supernatants was regarded as cell-free NETs, while extracellular DNA on culture wells was regarded as anchored NETs. SYTOX Green detected NETs was significantly higher in culture supernatants than that of control in human (A-i) and murine leukocytes (A-ii). Anchored NETs on culture wells after removal of culture supernatants were significantly higher than that of control wells in human (B-i) and murine leukocytes (B-ii). In fluorescence microscopic examination (C), SYTOX Green stained both extracellular DNAs that were anchored to neutrophils (anchored NETs; green) and nuclei of non-viable neutrophils (green). The data were obtained from representative results of at least three independently repeated experiments, and presented as mean+standard error. *P<0.05, and **P<0.01 versus control, respectively.
(TIF)

Figure S2 **Morphological characteristics of NETs** ***ex vivo*** **.** Murine leukocytes obtained from GFP mice (5×10^5^ per well) were seeded onto 16-mm polylysine-coated coverslips in 12-well tissue culture plates. After stimulation with LPS (20 µg/mL) for 6 h, SYTOX Orange (A), Alexa Fluor 594-labeled anti-histone antibody (B), and Alexa Fluor 594-labeled anti-NE antibody (C) was used for the detection of NETs. Ex vivo leukocytes (large, round cells; green), platelets (smaller ones; green), and NETs (red) were observed. Ex vivo NETs were characterized as linear structures (A-i, B-i, and C-i), reticular structures anchored to leukocytes (A-ii, B-ii, and C-ii), reticulolinear structures anchored to leukocytes (A-iii, and B-iii), membranous structures on the surface of leukocytes (C-iii), and spot-like structures anchored to leukocytes (A-iv, B-iv, and C-iv). With regard to the detection of ex vivo NETs, SYTOX Orange, anti-histone antibody, and anti-NE antibody showed nearly same ability to stain NETs, respectively. However, SYTOX orange stained the nuclei of non-viable cells more often than the others.
(TIF)

Figure S3 **Plasma cell-free DNA in a murine sepsis model.** The intraperitoneal administration of LPS at a dose of 20 mg/kg induced severe septic condition for mice. Heparinized blood was obtained from normal control (n = 5) and LPS-treated mice (5–10 mice at each time point). The concentration of Plasma DNA was quantified using a Quant-iTTM PicoGreen dsDNA Assay Kit. Plasma DNA was significantly increased in LPS-treated mice compared with normal control mice with a peak at 24 h after LPS administration. Data was presented as mean+standard error. *P<0.05 versus control.
(TIF)

Figure S4 **Leukocytes showing cytoplasmic vacuoles.** Leukocytes showing cytoplasmic vacuoles were observed in LPS-treated mice at the subcellular level (i, ii; arrows). They were more frequently observed in postcapillary venules of the cecum than arterioles or hepatic sinusoids. Some of them released NETs (red) (iii, iv; arrows).
(TIF)

Figure S5 **Neutrophil recruitment and endothelial injury in LPS treated mice.** Neutrophil infiltration and endothelial injury in the liver and lung of LPS treated mice were evaluated by immunohistochemistry against Gr-1 and CD31, respectively. The number of Gr-1 positive neutrophils was significantly higher in both liver (p<0.01) and lung (p<0.01) of LPS-treated mice than normal control mice, respectively. The score of endothelial integrity based on the percentage of CD31 positive endothelial cells was significantly lower in the lung (p<0.05) than normal control mice. The score of endothelial integrity was lower in the liver than normal control mice, but the difference was not statistically significant (p = 0.057). Data was presented as mean+standard error. *P<0.05, and **P<0.01 versus control, respectively.
(TIF)

Movie S1 **Anchored NETs (reticular form) in postcapillary venules of the cecum.**
(MP4)

Movie S2 **Anchored NETs (linear form) in postcapillary venules of the cecum.**
(MP4)

Movie S3 **Anchored NETs (spot-like form) in in hepatic sinusoids of the liver.**
(MP4)

Movie S4 **Cell-free DNA in in hepatic sinusoids of the liver.**
(MP4)

Movie S5 **Circulating cell-free DNA (cotton-like form) in postcapillary venules of the cecum.**
(MP4)

Movie S6 **The binding of platelet aggregates to cell-free NETs in the venules.**
(MP4)

Movie S7 **The adhesion of cell-free NETs to vascular endothelium in the arterioles.**
(MP4)

Movie S8 **The leukocyte with cytoplasmic vacuoles and NETs.**
(MP4)

Movie S9 **Platelet aggregates and leukocyte-platelet aggregates in postcapillary venules.**
(MP4)
