# Supplementary figures and images for: In Vivo Characterization of Neutrophil Extracellular Traps in Various Organs of a Murine Sepsis Model
Source: PLoS One. 2014 Nov 5;9(11):e111888. doi: 10.1371/journal.pone.0111888 (PMC4221155; doi:10.1371/journal.pone.0111888)

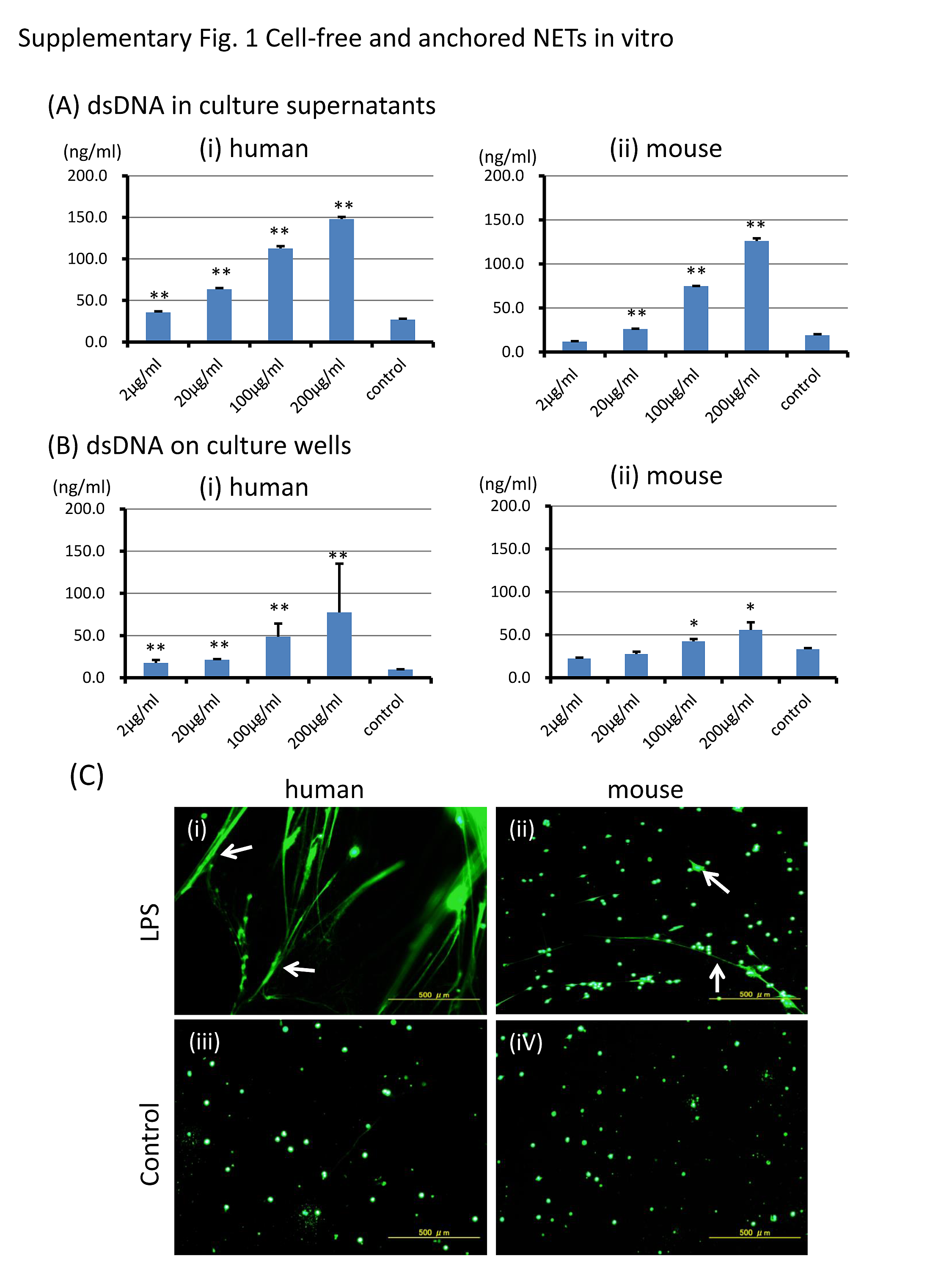

Supplement: Figure S1 — Cell-free and anchored NETs in vitro . NETs were classified as two forms: NETs that are released away from neutrophils (cell-free NETs), and those that are anchored to neutrophils (anchored NETs). In vitro study, extracellular DNA in culture supernatants was regarded as cell-free NETs, while extracellular DNA on culture wells was regarded as anchored NETs. SYTOX Green detected NETs was significantly higher in culture supernatants than that of control in human (A-i) and murine leukocytes (A-ii). Anchored NETs on culture wells after removal of culture supernatants were significantly higher than that of control wells in human (B-i) and murine leukocytes (B-ii). In fluorescence microscopic examination (C), SYTOX Green stained both extracellular DNAs that were anchored to neutrophils (anchored NETs; green) and nuclei of non-viable neutrophils (green). The data were obtained from representative results of at least three independently repeated experiments, and presented as mean+standard error. *P<0.05, and **P<0.01 versus control, respectively. (TIF) [file pone.0111888.s001.tif]

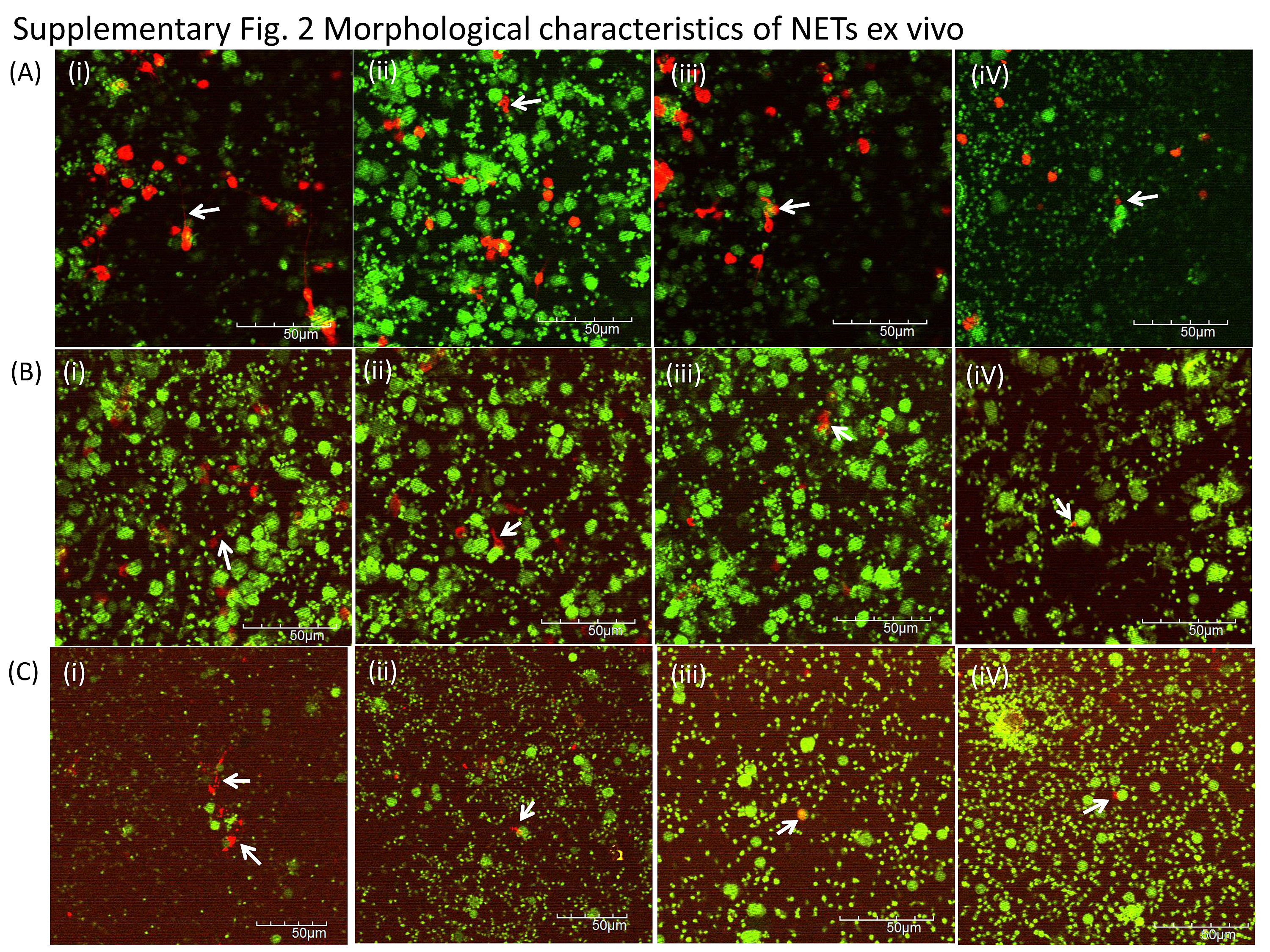

Supplement: Figure S2 — Morphological characteristics of NETs ex vivo . Murine leukocytes obtained from GFP mice (5×10^5^ per well) were seeded onto 16-mm polylysine-coated coverslips in 12-well tissue culture plates. After stimulation with LPS (20 µg/mL) for 6 h, SYTOX Orange (A), Alexa Fluor 594-labeled anti-histone antibody (B), and Alexa Fluor 594-labeled anti-NE antibody (C) was used for the detection of NETs. Ex vivo leukocytes (large, round cells; green), platelets (smaller ones; green), and NETs (red) were observed. Ex vivo NETs were characterized as linear structures (A-i, B-i, and C-i), reticular structures anchored to leukocytes (A-ii, B-ii, and C-ii), reticulolinear structures anchored to leukocytes (A-iii, and B-iii), membranous structures on the surface of leukocytes (C-iii), and spot-like structures anchored to leukocytes (A-iv, B-iv, and C-iv). With regard to the detection of ex vivo NETs, SYTOX Orange, anti-histone antibody, and anti-NE antibody showed nearly same ability to stain NETs, respectively. However, SYTOX orange stained the nuclei of non-viable cells more often than the others. (TIF) [file pone.0111888.s002.tif]

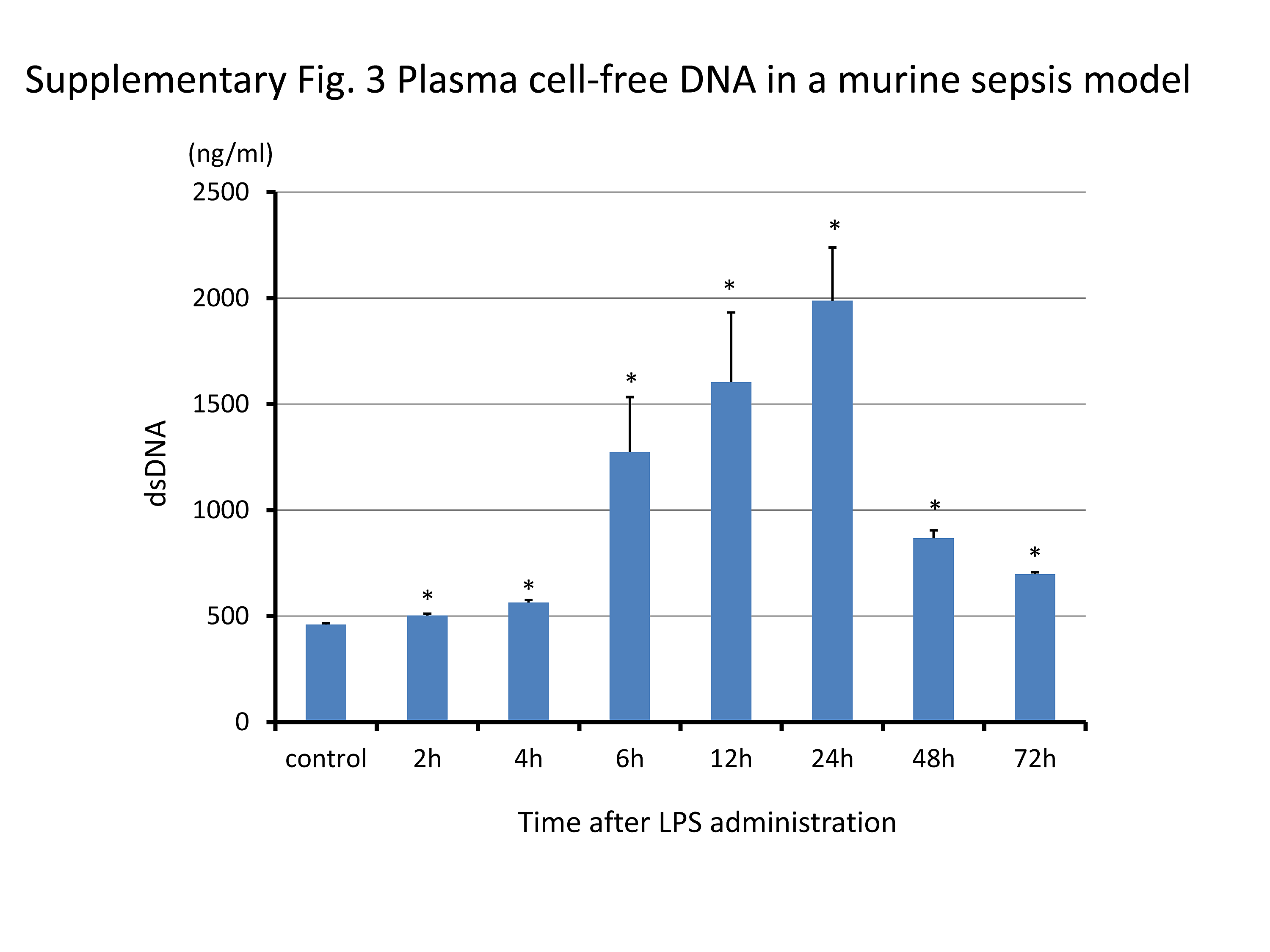

Supplement: Figure S3 — Plasma cell-free DNA in a murine sepsis model. The intraperitoneal administration of LPS at a dose of 20 mg/kg induced severe septic condition for mice. Heparinized blood was obtained from normal control (n = 5) and LPS-treated mice (5–10 mice at each time point). The concentration of Plasma DNA was quantified using a Quant-iTTM PicoGreen dsDNA Assay Kit. Plasma DNA was significantly increased in LPS-treated mice compared with normal control mice with a peak at 24 h after LPS administration. Data was presented as mean+standard error. *P<0.05 versus control. (TIF) [file pone.0111888.s003.tif]

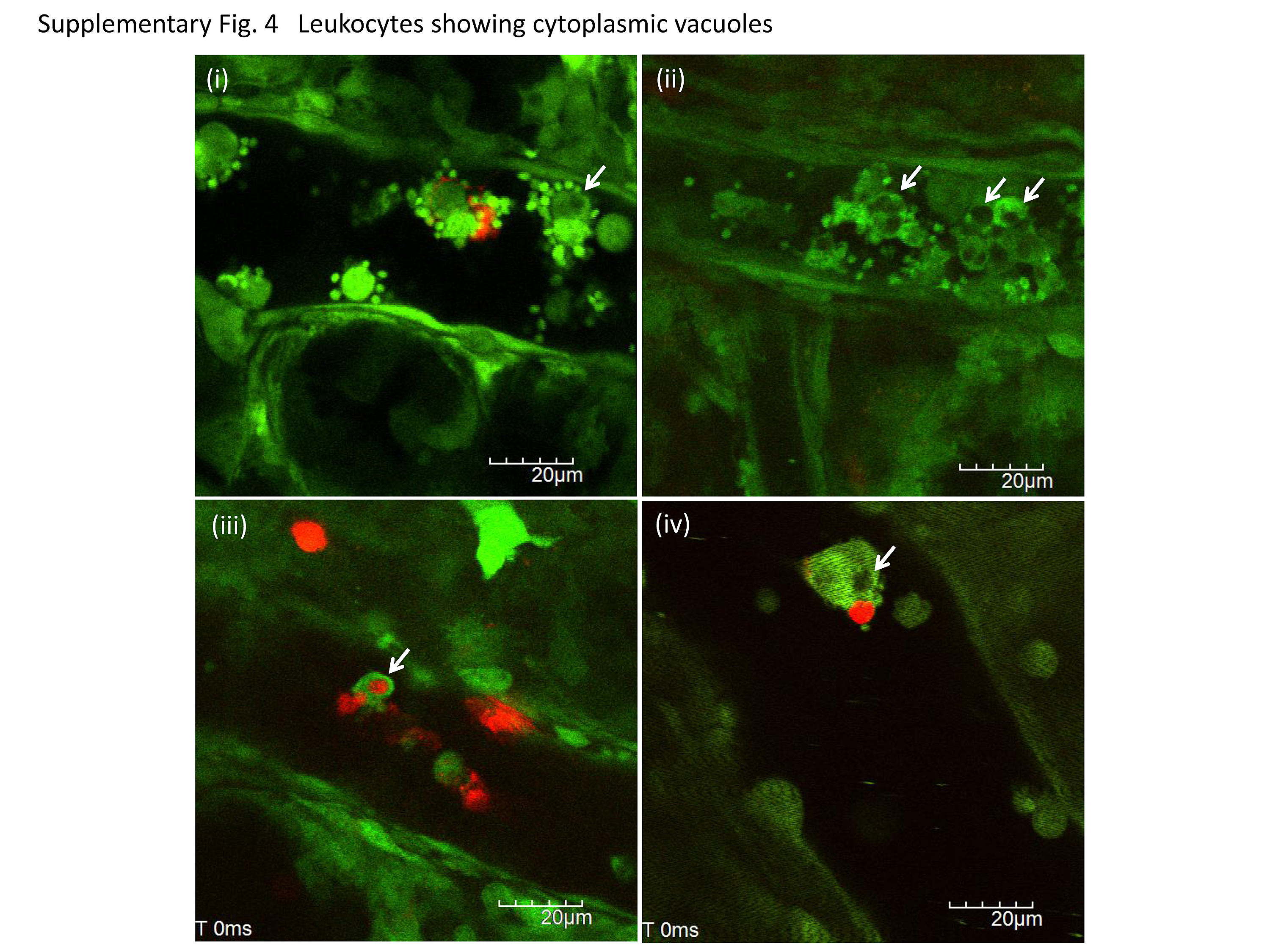

Supplement: Figure S4 — Leukocytes showing cytoplasmic vacuoles. Leukocytes showing cytoplasmic vacuoles were observed in LPS-treated mice at the subcellular level (i, ii; arrows). They were more frequently observed in postcapillary venules of the cecum than arterioles or hepatic sinusoids. Some of them released NETs (red) (iii, iv; arrows). (TIF) [file pone.0111888.s004.tif]

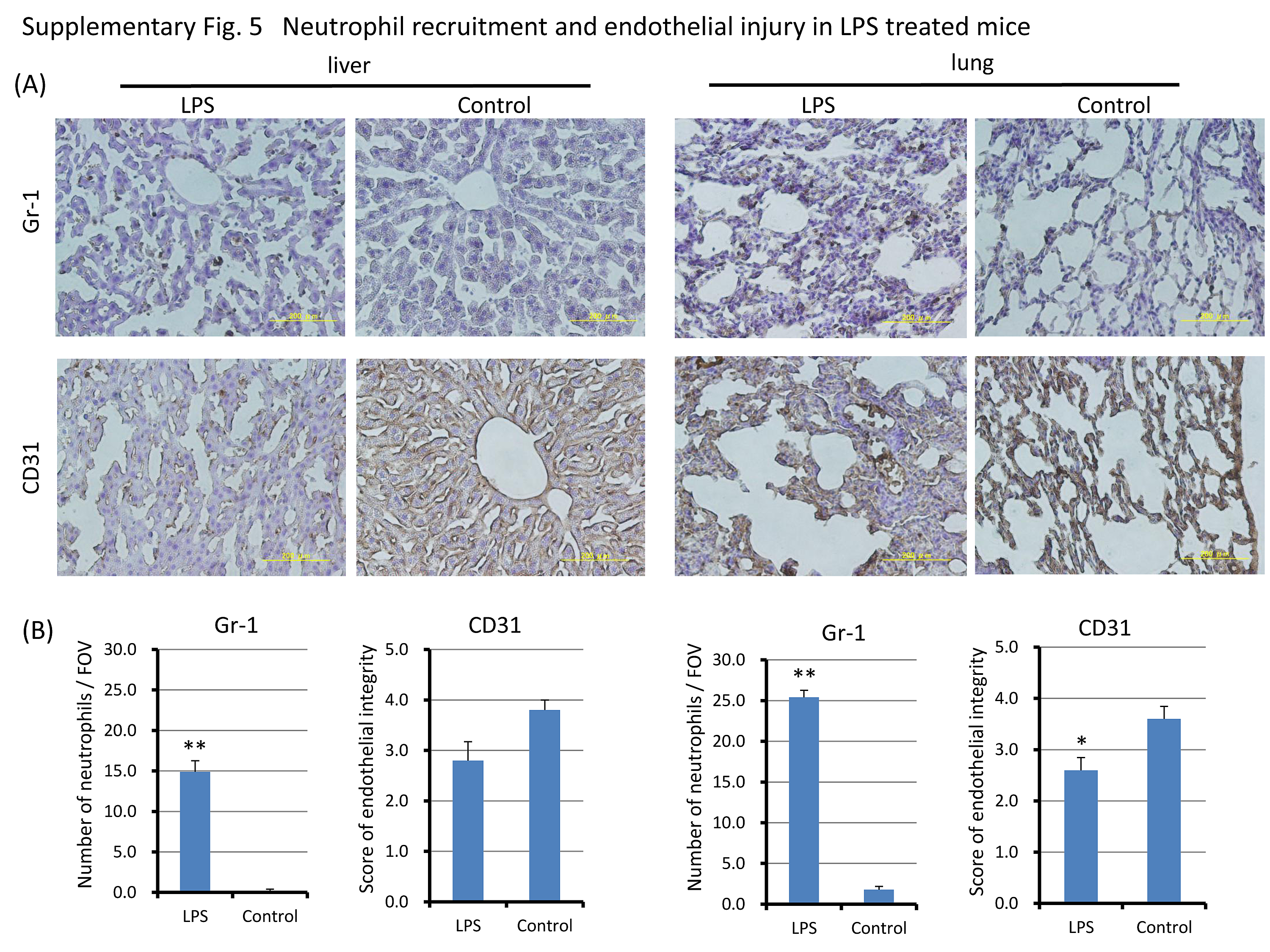

Supplement: Figure S5 — Neutrophil recruitment and endothelial injury in LPS treated mice. Neutrophil infiltration and endothelial injury in the liver and lung of LPS treated mice were evaluated by immunohistochemistry against Gr-1 and CD31, respectively. The number of Gr-1 positive neutrophils was significantly higher in both liver (p<0.01) and lung (p<0.01) of LPS-treated mice than normal control mice, respectively. The score of endothelial integrity based on the percentage of CD31 positive endothelial cells was significantly lower in the lung (p<0.05) than normal control mice. The score of endothelial integrity was lower in the liver than normal control mice, but the difference was not statistically significant (p = 0.057). Data was presented as mean+standard error. *P<0.05, and **P<0.01 versus control, respectively. (TIF) [file pone.0111888.s005.tif]
